# Self-management of patients with type 2 diabetes in Chaoyang District, Beijing, and challenges in utilizing family and community resources: a mixed-methods study

**DOI:** 10.1186/s12875-025-03111-6

**Published:** 2025-11-24

**Authors:** Zi-Cheng Qi, Mei-Rong Wang, Yu Kang, Chen-Mei Zhao, Yan-Hong Wang, Juan Gao, Xiao-Juan Wang

**Affiliations:** 1 Department of General Practice, Beijing Chao-yang Hospital, Capital Medical University, 8 Gongren Tiyuchang Nanlu, Chaoyang District, Beijing, 100020 China; 2 Sanlitun Community Health Service Center, Chaoyang District, Beijing, 100027 China; 3 Liulitun Community Health Service Center, Chaoyang District, Beijing, 100026 China; 4 Gaobeidian Community Health Service Center, Chaoyang District, Beijing, 100024 China

**Keywords:** Type 2 diabetes mellitus, Self-Management, Community health services, Family support, Long-term diabetes management

## Abstract

**Background:**

The prevalence of type 2 diabetes mellitus (T2DM) in China has been steadily increasing, making it a major public health concern. Effective self-management, along with family and community support, is critical for disease control and the prevention of complications. However, many patients continue to face challenges in disease management. In addition to requiring proactive and standardized diabetes care from health service providers, patients also need to strengthen their self-management skills and mobilize family/community support resources. Further investigation into these challenges and potential solutions is warranted. This study aims to systematically evaluate the self-management behaviors of patients with T2DM and the collaborative management capacity of family caregivers in Chaoyang District, Beijing. It also intends to explore the practical challenges faced by community-based T2DM patients in long-term disease management with respect to self-management, family support, and community support. In addition, it seeks to identify optimization pathways for health management models based on family support systems, thereby providing both theoretical grounding and practical reference for developing comprehensive intervention strategies under the concept of proactive health.

**Methods:**

An explanatory sequential design was employed in mixed methods research. This study first conducted a cross-sectional survey to assess the self-management status of T2DM patients and their caregivers attending medical institutions. Subsequently, qualitative research was employed to explore the specific challenges faced by community-based T2DM patients in long-term disease management, particularly regarding self-management, family support, and community support. A cross-sectional survey of T2DM patients and their caregivers was conducted in four healthcare institutions in Chaoyang District, Beijing, using standardized self-management questionnaires, and quantitative data were collected from June to October 2024. Semi-structured interviews were carried out with purposively selected patients, and qualitative interviews were conducted between April and June 2025. Quantitative data were analyzed descriptively, and qualitative data were thematically analyzed using Colaizzi’s method. Methodological triangulation was adopted to cross-validate findings obtained from the cross-sectional survey and semi-structured interviews.

**Results:**

Among 206 T2DM patients, attitude scores were highest, knowledge moderate, and practice lowest. The highest mean score of patient’s knowledge dimension was observed for blood pressure and blood glucose knowledge (88.84 ± 27.93), while the lowest was basic knowledge (60.68 ± 32.29), At the practice level, foot care had only a moderate score (69.12 ± 21.33), only 22.33% performing it regularly. Exercise scored lowest (67.48 ± 29.85), with 38.35% of patients exercising regularly. Among 125 caregivers, attitudes were favorable, but notable deficiencies persisted across diabetes-related knowledge and other domains. The highest mean scores of knowledge dimension were observed for blood pressure/glucose knowledge (93.87 ± 17.65), While the lowest scores were for basic knowledge (74.93 ± 29.83). Qualitative findings from 25 patients revealed barriers including weak self-management beliefs, poor self-control, and limited knowledge, as well as inadequate family support and gaps in individualized, continuous, and effective community services. Qualitative data further revealed emotional and stress-related challenges that affected patients’ adherence and coping capacity.

**Conclusion:**

Patients with T2DM in Chaoyang District, Beijing demonstrated notable deficiencies in disease management knowledge and low adherence to key self-management behaviors. Although caregivers performed better overall, their capacity for support also requires strengthening. Barriers at multiple levels may hinder effective glycemic control and make achievement of management goals challenging.

## Background

Type 2 diabetes mellitus (T2DM) is a chronic metabolic disease characterized by insulin resistance and relative insulin deficiency. In China, the prevalence of diabetes has risen rapidly due to population aging [1, 2]and lifestyle changes such as sedentary behavior, unhealthy diets, and physical inactivity [3]. The national prevalence has reached 11.9% (12.4% using the ADA 2010 criteria), with T2DM being the predominant type[4]. In 2021, China had approximately 140 million people with diabetes, accounting for nearly one quarter of the global burden, and this number is projected to increase to 174 million by 2045, ranking first worldwide [5]. The growing prevalence and enormous patient population have generated a substantial disease burden. However, the awareness, treatment, and control rates of diabetes have not improved significantly and remain relatively low [4]. Similar to many other middle-income countries, China has only relatively recently recognized the importance of social determinants of health and begun to address urban–rural health inequities [6]. In response, the *Healthy China Initiative (2019–2030)* listed diabetes prevention and control among its 15 key actions [7], highlighting the essential role of primary healthcare institutions in treatment and management, and calling for effective interventions to slow disease progression, improve quality of life, and extend healthy life expectancy.

Long-term diabetes management requires the joint participation of patients, families, and communities. Effective self-management behaviors—including diet control, regular exercise, medication adherence, and blood glucose monitoring—are critical to disease control and prevention of complications. However, studies show that community patients often struggle with barriers such as a lack of knowledge about diabetes self-management practices, cultural practices, and lack of guidelines and protocols for counselling [8, 9]. Families, as the most direct support system in daily life, also play a vital role. Evidence suggests that stronger family involvement is beneficial to the control of T2DM [10]. Yet, in practice, the potential of family support is often underutilized. Community health institutions are also crucial, providing health education, chronic disease follow-up, and behavioral interventions. Social support also had a moderating role in the link between diabetes distress and resilience [11]. Effective community-based programs can improve the quality of life and self-management ability of older patients with comorbid T2DM without increasing total healthcare costs [12]. Nevertheless, community support remains limited, often lacking diversity and proactivity, and fails to fully meet patient needs.

China is currently exploring an integrated management pathway to address the above challenges. Management strategies, including the family doctor contract service model, have effectively increased the utilization of primary healthcare services and improved glycemic control [13–15]. However, gaps remain in selecting optimal care strategies and achieving sustained management outcomes [15]. One important reason may be the lack of a clear understanding of T2DM patients’ current status and practical challenges in self-management, family, and community support, leading to insufficiently targeted interventions. Therefore, this study aimed to systematically assess the current status and barriers at the patient, family, and community levels and to provide evidence to optimize comprehensive, family-centered interventions within the framework of proactive health.

## Methods

We used an explanatory sequential design in mixed methods research.

### Phase 1: a cross-sectional survey on self-management and family support among patients with T2DM

This phase utilized a questionnaire-based survey to assess diabetic patients’ knowledge, self-management behaviors, disease management attitudes, and caregivers’ supportive attitudes and knowledge. The questionnaire consisted of a demographic section capturing participants’ gender, age, education, lifestyle habits, and diabetes monitoring practices, and a 35-item simplified Knowledge-Attitude- Practice (KAP) scale adapted from the Chinese Center for Disease Control and Prevention [16]. The KAP scale evaluated three domains: knowledge (15 true/false items, 1 point/correct answer), attitude (5 Likert-scale items scored 0.2-1), and behavior (11 Likert-scale items scored 0.2-1 and 4 ternary-response items). The modified scale demonstrated acceptable reliability (Cronbach’s α = 0.775, all subscales >0.70). The reliability analysis of the assessment scales in presented in Table 1. Scores were standardized to a 100-point scale using the formula: (actual score - minimum theoretical score)/(maximum - minimum theoretical score) *100, with performance categorized as good (≥ 85), moderate (60–84), or poor (< 60).

**Table 1:** Reliability analysis of assessment scales (Cronbach’s α Coefficients)

Scale/Subscale	Group	Cronbach’s α
KAP Scale	Patient	0.775
Knowledge Subscale	Patient	0.796
Attitude Subscale	Patient	0.947
Practice Subscale	Patient	0.717
Knowledge Subscale	Caregiver	0.755
Attitude Subscale	Caregiver	0.975

From June to October 2024, one tertiary hospital and three community health service centers within its urban medical consortium in Chaoyang District, Beijing, were selected. General practice teams recruited patients with T2DM during routine diagnosis, treatment, and follow-up management. Eligible patients and their caregivers were assigned identification numbers, and a simple random sampling method with a random number table was used to select participants. Based on prior studies [17] and an estimated 90% questionnaire response rate, the planned sample size was approximately 120 patients and 120 caregivers. Written informed consent was obtained prior to participation.

Inclusion criteria: Patients aged ≥ 18 years with T2DM diagnosed according to the 2020 Chinese Guidelines for the Prevention and Treatment of T2DM [18], and their caregivers; participants with clear consciousness, normal cognitive function, and no communication barriers; patients receiving rehabilitation or convalescent care at home, registered at a community health service center, and under physician management for >1 month; caregivers who had lived with patients ≥ 2 h daily for at least 6 months.

Exclusion criteria: Patients with severe complications, those unable to regularly attend community health services, or those unwilling to participate.

Data were collected through face-to-face questionnaires with the cooperation of staff from the participating community health centers. For eligible participants, trained investigators distributed the questionnaires on site. Participants completed the questionnaires independently; if physical or educational limitations prevented self-completion, investigators read the items aloud and recorded participants’ self-reported responses. All questionnaires were collected immediately, checked for completeness, and verified to ensure authenticity and validity.

All data were double-entered and verified using *EpiData 3.1* and analyzed with *SPSS version 25.0*. Continuous variables were expressed as mean ± standard deviation ($\:\stackrel{-}{x}\pm\:s$) and categorical variables as frequencies and percentages. Statistical significance was defined as *P* < 0.05. Normality of data distribution was assessed using the *Kolmogorov–Smirnov test* (or the *Shapiro–Wilk test* when sample size < 200). For normally distributed data, *t*-tests or *one-way ANOVA* were applied, followed by *Bonferroni-adjusted post hoc tests* when appropriate. For non-normally distributed data, the *Mann–Whitney U test* was used for independent samples, while the *Wilcoxon signed-rank test* was used for paired data; the *Friedman test* was applied for repeated measures.

### Phase 2: qualitative study on disease management and barriers to family and community resource utilization in patients with T2DM

Semi-structured interviews were conducted for the qualitative study. An interview guide was first developed based on the study objectives through literature review and team discussions. The draft guide was then reviewed by general practitioners and endocrinology experts, who provided feedback and suggestions. Prior to the formal interviews, two T2DM patients were invited for pilot interviews to test the quality of the guide. Based on their feedback, the wording and structure of questions were revised to ensure clarity, comprehensiveness, and depth. The final interview guide is presented in Table 2.

**Table 2:** Interview guide

Perspective	Analytical Focus	Sample Interview Questions
Self-management	Measures taken for self-management	In your daily life, do you actively take any measures or make changes to prevent or control diabetes?
	Beliefs about self-management efficacy	Do you feel you have the ability to manage diabetes well and reduce its complications?
	Difficulties in self-management	What difficulties or challenges do you face in managing diabetes?
Family support	Current family support	What kind of support do your family members provide for your diabetes management?
	Family support needs	In what ways do you expect your family to help you with diabetes management?
Community support	Community support needs	What diabetes-related health promotion services do you expect to receive from the community?
	Suggestions for improving community services	What improvements should be made in the diabetes services provided by your community health center?

From April to June 2025, purposive sampling was used to recruit T2DM patients who had participated in the cross-sectional survey and were receiving care in general practice departments of community health service centers. The inclusion and exclusion criteria were consistent with those previously described. The sample size was determined based on the principle of data saturation, defined as the point at which no new significant information emerged from interviews and no new codes or themes appeared during data analysis. All participants provided written informed consent prior to the interviews.

Using a pre-developed interview guide, one-on-one semi-structured interviews were conducted with T2DM patients. After obtaining informed consent, interview appointments were scheduled. Interviews were held in private meeting rooms at the community health centers to ensure a quiet, undisturbed environment. Two interviewers trained in qualitative methodology conducted the interviews. Before each interview, participants were informed about the study’s purpose, procedures, and significance, and written consent for audio recording was obtained with assurance of confidentiality. During the interviews, questions were flexibly adapted based on the guide. Each session lasted approximately 30–60 min, and participants were thanked upon completion.

Within 24 h after each interview, audio recordings were transcribed verbatim and returned to participants for member checking. Transcripts were managed using *NVivo 15.0*. Two researchers independently analyzed the data following *Colaizzi’s seven-step method*, involving repeated reading, coding, categorization, and theme extraction. Discrepancies were discussed and resolved through consultation with other members of the research team until consensus on the final themes was reached.

To ensure the trustworthiness of the study and enhance the credibility and validity of the findings, methodological triangulation was employed by integrating quantitative and qualitative data.

## Result

### Phase 1 a cross-sectional survey on self-management and family support among patients with T2DM

A total of 209 questionnaires were collected from patients at Beijing Chaoyang Hospital, Capital Medical University, Sanlitun Community Health Service Center, Liulitun Community Health Service Center, and Gaobeidian Community Health Service Center. Three questionnaires with > 10% missing items were excluded, leaving 206 questionnaires for analysis, with a response rate of 98.6%. The patients ranged in age from 30 to 90 years. Demographic characteristics are presented in Table 3.

**Table 3:** Demographic information of patients with T2DM (*n* = 206)

Item	Category	Number (*n*)	Proportion (%)	KAP scale score$\:(\stackrel{-}{x}\pm\:s)$	*P*-value
Healthcare Institution	Beijing Chaoyang Hospital	66	32.04	74.32 ± 13.66	<0.001
	Sanlitun Health Service Center	48	23.30	76.48 ± 12.32	
	Liulitun Health Service Center	62	30.10	84.33 ± 8.30	
	Gaobeidian Health Service Center	30	14.56	72.41 ± 9.04	
Gender	Male	108	52.43	78.91 ± 11.32	0.094
	Female	98	47.57	76.07 ± 12.88	
Age	< 45	12	5.83	81.50 ± 10.10	0.474
	45–60	57	27.67	77.40 ± 10.98	
	≥ 60	137	66.50	77.28 ± 12.76	
Education Level	Primary or below	19	9.22	70.47 ± 13.71	0.014
	Junior/Technical school	45	21.84	75.85 ± 12.19	
	High school	73	35.44	77.83 ± 10.46	
	College	44	21.36	80.62 ± 10.86	
	Bachelor’s or above	25	12.14	79.85 ± 15.44	
Occupation	Professional technical staff	30	14.56	84.55 ± 7.45	<0.001
	State/government agencies and institutions	20	9.71	76.70 ± 10.31	
	Business/service personnel	3	1.46	83.65 ± 13.45	
	Self-employed	25	12.14	77.31 ± 10.03	
	Unemployed	3	1.46	72.33 ± 9.26	
	Retired	90	43.69	78.66 ± 12.02	
	Other	35	16.99	69.34 ± 13.98	
Smoking History	Currently smoking	36	17.48	75.89 ± 11.23	0.519
	Never smoked	130	63.11	78.38 ± 11.91	
	Quit smoking	37	17.96	77.35 ± 11.91	
	Exposed to secondhand smoke	3	1.46	64.57 ± 29.37	
Drinking History	Never drank	140	67.96	78.60 ± 12.37	0.056
	Quit drinking	27	13.11	72.37 ± 13.51	
	Currently drinking	39	18.93	77.42 ± 9.37	

Scores on the Knowledge, Attitude, and Practice (KAP) scale were compared across seven demographic variables (healthcare institution, sex, age, education level, occupation, smoking history, and drinking history). Significant differences were observed by healthcare institution, education level, and occupation (*P* < 0.05). Patients from different institutions scored differently, reflecting variations in patient characteristics and the quality of health education provided. Higher education was associated with higher KAP scores, and patients employed in technical, commercial, or service occupations also had higher scores. No significant differences were found by sex, age, smoking, or drinking history (*P* > 0.05).

Among 206 T2DM patients, the mean score on the KAP scale was 77.56 ± 12.14, indicating a moderate level (range: 30.82–98.74). The highest subscale scores were observed for attitude (92.28 ± 13.36; maximum 100), suggesting that most patients had a positive attitude toward disease management. Knowledge scores were moderate (77.38 ± 19.52), with only 48.6% of patients demonstrating good mastery of diabetes-related knowledge. Practice scores were lowest (73.17 ± 16.15; minimum 20.31), with only 19.4% of patients reaching a good level, highlighting a substantial gap in self-management behaviors. Detailed scores are presented in Table 4.

**Table 4:** Scores of KAP scale and subscales in patients with T2DM

Item	Score $\:(\stackrel{-}{\text{x}}\pm\:\text{s})$	Score level(%)		
		≥ 85	≥ 60, <85	<60
Knowledge Subscale	77.38 ± 19.52	48.6	36.4	15.0
Attitude Subscale	92.28 ± 13.36	75.7	22.8	1.5
Practice Subscale	73.17 ± 16.15	19.4	62.6	18.0
KAP Scale	77.56 ± 12.14	36.9	63.1	-

The scoring levels are classified into three categories: good, medium, and poor. A score ≥ 85 is defined as good; ≥60 but < 85 as medium; and < 60 as poor

Scores for the different dimensions of the KAP knowledge subscale are presented in Table 5. Among 206 patients, the highest mean score was observed for blood pressure and blood glucose knowledge (88.84 ± 27.93), while the lowest was basic knowledge (60.68 ± 32.29), with only 26.61% of patients demonstrating adequate reserves in this area. The *Friedman test* was used to compare scores across dimensions and showed significant overall differences (*P* < 0.05). Pairwise comparisons were performed using the *Wilcoxon signed-rank test* for related samples. After *Bonferroni correction* for multiple comparisons (α = 0.05/15 ≈ 0.003), no significant differences were found among blood pressure/glucose, medication, and dietary knowledge. However, basic knowledge scores were significantly lower than all other dimensions (*P* < 0.003). Hypoglycemia self-rescue knowledge was significantly lower than blood pressure/glucose, medication, and dietary knowledge (*P* < 0.003), and exercise knowledge was significantly lower than blood pressure/glucose and diet knowledge (*P* < 0.003).

**Table 5:** Scores of knowledge subscale in patients with T2DM

Dimensions of Knowledge	Theoretical Range	Min Value	Max Value	Score $\:(\stackrel{-}{x}\pm\:s)$	*P*-value
Blood Pressure and Blood Sugar	0–3	0	3	88.84 ± 27.93	<0.05
Medication	0–1	0	1	84.95 ± 35.84	
Dietary	0–2	0.6	2	83.74 ± 30.29	
Exercise	0–3	0	3	78.48 ± 29.55	
Hypoglycemia Self-Rescue	0–3	0	3	74.76 ± 21.06	
Basic	0–3	0	3	60.68 ± 32.29	
Total Score	0–15	1	15	77.38 ± 19.52	

Scores for different dimensions of the attitude subscale are shown in Table 6. Patients demonstrated relatively high and comparable scores across all dimensions. *Friedman test* results indicated no statistically significant differences among the dimensions (*P* > 0.05).

**Table 6:** Scores of the attitude subscale in patients with T2DM

Dimensions of Attitude	Theoretical Range	Min Value		Max Value		Score $\:(\stackrel{-}{x}\pm\:s)$	*P*-value
Health Education	0.2–1		0.2		1	0.934 ± 0.124	> 0.05
Dietary Control	0.2–1		0.2		1	0.934 ± 0.124	
Blood Glucose Monitoring	0.2–1		0.2		1	0.940 ± 0.116	
Medication Use	0.2–1		0.2		1	0.950 ± 0.103	
Exercise	0.2–1		0.2		1	0.940 ± 0.118	

Scores for the different dimensions of the practice subscale are presented in Table 7. Among 206 patients, the highest mean score was observed for medication adherence (85.19 ± 24.05), followed by dietary and blood pressure/glucose monitoring. Foot care had only a moderate score (69.12 ± 21.33), with only 22.33% performing it regularly, while exercise was lowest (67.48 ± 29.85), with 38.35% of patients exercising regularly. The *Friedman test* showed significant overall differences among the dimensions (*P* < 0.05). Pairwise comparisons using the *Wilcoxon signed-rank test* for related samples with *Bonferroni correction* (α = 0.05/15 ≈ 0.003) indicated that exercise scores were significantly lower than medication, diet, and blood pressure/glucose monitoring (*P* < 0.003), and foot care scores were significantly lower than medication and diet (*P* < 0.003).

**Table 7:** Scores of the practice subscale in patients with T2DM

Dimensions of Practice	Theoretical Range	Min Value	Max Value	Score $\:(\stackrel{-}{\text{x}}\pm\:\text{s})$	*P*-value
Medication Adherence	0.2–1	0.2	1	85.19 ± 24.05	<0.05
Dietary	0.6–3	1	3	76.98 ± 25.07	
Blood Pressure and Blood Glucose Monitoring	0.6–3	0.6	3	74.39 ± 28.31	
Complication Management	0–3	0	3	72.49 ± 35.74	
Foot Care	0.4–3	0.6	3	69.12 ± 21.33	
Exercise	0.4–2	0.6	2	67.48 ± 29.85	
Total Score	2.2–15	2.6	11	73.17 ± 16.15	

A total of 125 questionnaires were collected from caregivers of T2DM patients at Sanlitun, Liulitun, and Gaobeidian Community Health Service Centers in Chaoyang District, Beijing. All 125 questionnaires were valid, yielding a 100% response rate. The caregivers ranged in age from 20 to 82 years, with a mean age of 54.3 ± 14.38 years. Demographic characteristics are presented in Table 8.

**Table 8:** Demographic information of caregivers (*n* = 125)

Item	Category	Number (*n*)	Proportion (%)	KAB scale score$\:(\stackrel{-}{\text{x}}\pm\:\text{s})$	*P*-value
Healthcare Institution	Sanlitun Health Service Center	46	36.80	86.77 ± 17.09	0.01
	Liulitun Health Service Center	49	39.20	88.40 ± 11.68	
	Gaobeidian Health Service Center	30	24.00	80.00 ± 14.05	
Gender	Male	51	40.80	84.44 ± 13.52	0.223
	Female	74	59.20	86.71 ± 15.49	
Age	< 45	37	29.60	83.33 ± 14.15	0.237
	45–60	34	27.20	85.79 ± 19.69	
	≥ 60	54	43.20	87.47 ± 11.00	
Education Level	Primary or below	5	4.00	76.84 ± 17.69	0.418
	Junior school	22	17.60	83.73 ± 13.09	
	High school	31	24.80	87.27 ± 11.23	
	Junior college	31	24.80	86.99 ± 13.01	
	Bachelor’s or above	36	28.80	85.96 ± 18.88	
Occupation	Professional technical staff	25	20.00	89.31 ± 13.50	0.035
	State/government agencies and institutions	16	12.80	91.12 ± 8.53	
	Business/service personnel	13	10.40	80.97 ± 25.67	
	Self-employed	6	4.80	75.44 ± 16.19	
	Unemployed	3	2.40	75.44 ± 10.96	
	Retired	52	41.60	86.78 ± 11.61	
	Other	10	8.00	78.84 ± 16.48	
Relationship	Spouse	68	54.40	86.44 ± 11.72	0.997
	Children	42	33.60	84.76 ± 18.54	
	Siblings	1	0.80	89.47	
	Parents	10	8.00	85.89 ± 16.38	
	Other	4	3.20	84.21 ± 18.23	

Scores on the simplified diabetes knowledge and attitude scale were compared across six demographic variables (institution, sex, age, education level, occupation, and type of kinship). Significant differences were observed by institution and occupation (*P* < 0.05). Variations in caregiver scores reflected differences in individual background and the quality of institutional health education. Caregivers employed as technical professionals or government/public institution staff achieved higher scores. No significant differences were found by sex, age, kinship type, or education level (*P* > 0.05).

Among 125 caregivers of T2DM patients, the overall mean score on the simplified diabetes knowledge and attitude scale was 85.79 ± 14.70 (range: 0–100), with 58.4% classified as good and 35.2% as moderate. Knowledge scores were lowest, with a mean of 83.25 ± 16.67 and 60.8% achieving a good level. Attitude scores were highest, with a mean of 95.28 ± 12.38 (range: 0–100), and 84.0% at a good level, indicating that most caregivers demonstrated a positive attitude toward supporting patients in diabetes management. Detailed scores are presented in Table 9.

**Table 9:** Scores of diabetes knowledge and attitude scale for caregivers (*n* = 125)

Items	Score$\:(\stackrel{-}{x}\pm\:s)$	Score level (%)		
		≥ 85	≥ 60, <85	<60
Knowledge	83.25 ± 16.67	60.8	31.2	8.0
Attitude	95.28 ± 12.38	84.0	15.2	0.8
Total Score	85.79 ± 14.70	58.4	35.2	6.4

The scoring levels are classified into three categories: good, medium, and poor. A score ≥ 85 is defined as good; ≥60 but < 85 as medium; and < 60 as poor

Scores for the different dimensions of the caregivers’ knowledge subscale are presented in Table 10. The highest mean scores were observed for blood pressure/glucose knowledge (93.87 ± 17.65), dietary knowledge (86.40 ± 27.95), and medication knowledge (85.60 ± 35.25), all at a good level. The lowest scores were for basic knowledge (74.93 ± 29.83), which was at a slightly above moderate level. The *Friedman test* showed significant overall differences among the dimensions (*P* < 0.05). Pairwise comparisons using the *Wilcoxon signed-rank test* for related samples, with *Bonferroni correction* (α = 0.05/15 ≈ 0.003), indicated that blood pressure/glucose knowledge scores were significantly higher than those for basic knowledge, diet, exercise, and hypoglycemia self-rescue (*P* < 0.003).

**Table 10:** Scores of diabetes knowledge dimensions for caregivers

Dimensions of knowledge	Theoretical Range	Min Value	Max Value	Score $\:(\stackrel{-}{\text{x}}\pm\:\text{s})$	*P*-value
Blood Pressure and Blood Glucose	0–3	0	3	93.87 ± 17.65	<0.05
Dietary	0–2	0.6	2	86.40 ± 27.95	
Medication	0–1	0	1	85.60 ± 35.25	
Exercise	0–3	0	3	81.07 ± 25.16	
Hypoglycemia Self-Rescue	0–3	0	3	80.27 ± 24.72	
Basic Knowledge	0–3	0	3	74.93 ± 29.83	
Total Score	0–15	1	15	83.25 ± 16.67	

Scores for the different dimensions of the caregivers’ attitude subscale are shown in Table 11. Caregivers demonstrated consistently high scores across all attitude dimensions. The *Friedman test* indicated no statistically significant differences among the dimensions (*P* > 0.05).

**Table 11:** Scores of diabetes attitude dimensions for caregivers

Dimensions of Attitude	Theoretical Range	Min Value	Max Value	Score $\:(\stackrel{-}{\text{x}}\pm\:\text{s})$	*P*-value
Health Education	0.2–1.2	0.2	1	0.963 ± 0.10	> 0.05
Dietary Control	0.2–1.2	0.2	1	0.963 ± 0.10	
Blood Glucose Monitoring	0.2–1.2	0.2	1	0.958 ± 0.11	
Medication Use	0.2–1.2	0.2	1	0.966 ± 0.98	
Exercise	0.2–1.2	0.2	1	0.960 ± 0.11	

### Phase 2: qualitative study on disease management and barriers to family and community resource utilization in patients with T2DM

A total of 25 T2DM patients who were regularly managed by community health service centers were included in the qualitative study. Of these, 12 were male and 13 were female, with a mean age of 63.92 ± 10.73 years and a mean disease duration of 8.62 ± 7.51 years. Detailed information of the 25 respondents is presented in Table 12.

**Table 12:** Basic information of the 25 respondents

ID	Sex	Age(years)	Education Level	Occupation	Disease Duration (years)
N1	Male	76	Junior college	Retired	2
N2	Male	56	Junior college	Employed	11
N3	Male	83	Junior high	Retired	14
N4	Male	64	Senior high	Retired	19
N5	Female	58	Junior high	Farmer	20
N6	Male	54	Bachelor	Employed	3
N7	Male	84	Senior high	Retired	18
N8	Male	55	Junior high	Employed	2
N9	Female	80	Junior high	Retired	30
N10	Female	51	Bachelor	Employed	7
N11	Female	58	Junior college	Retired	3
N12	Male	56	Junior college	Employed	5
N13	Male	52	Junior college	Employed	3
N14	Female	58	Junior college	Retired	3
N15	Female	50	Bachelor	Employed	2
N16	Female	57	Junior college	Employed	4
N17	Female	71	Senior high	Retired	5
N18	Female	70	Primary school	Retired	10
N19	Male	68	Senior high	Retired	18
N20	Female	72	Junior high	Retired	11
N21	Female	62	Senior high	Retired	0.5
N22	Female	55	Junior college	Employed	1
N23	Female	82	Senior high	Retired	10
N24	Male	58	Junior college	Employed	8
N25	Male	68	Senior high	Retired	6

**Fig. 1:**
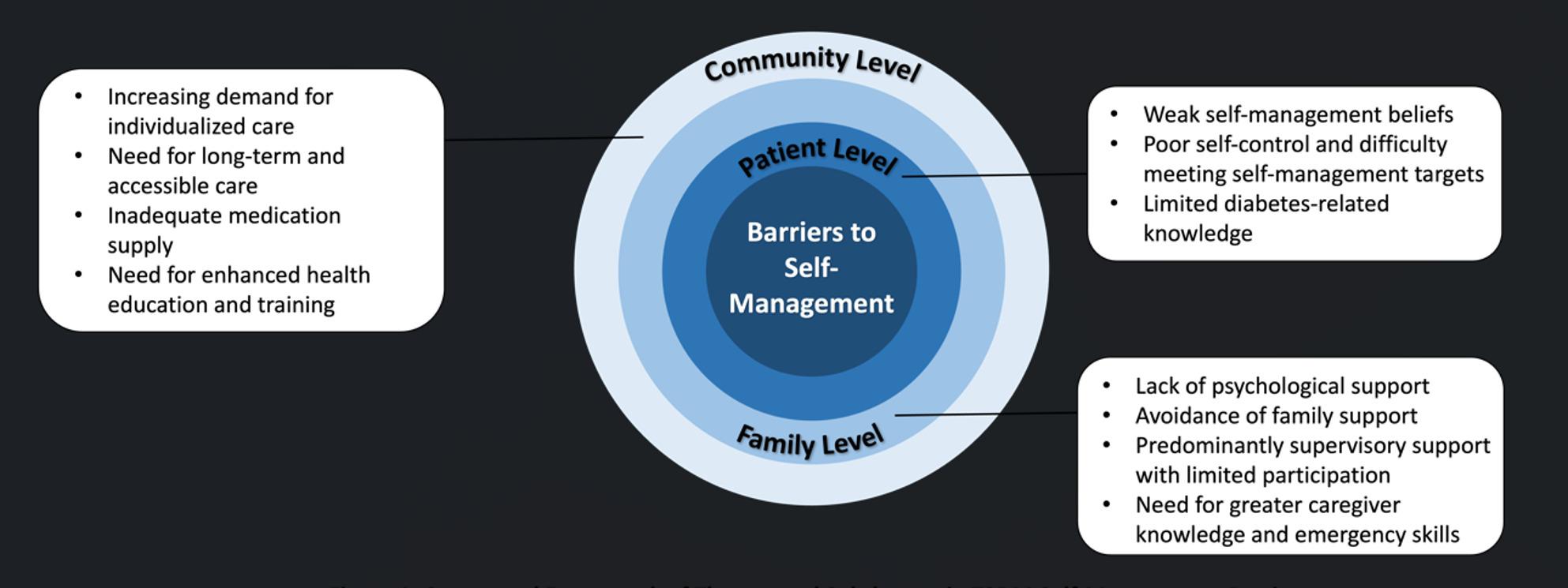
Conceptual Framework of Themes and Subthemes in T2DM Self-Management Barriers

Detailed qualitative results are shown in Table 13 and a conceptual framework of themes and subthemes in T2DM self-management barriers is showed in Fig. 1.

**Table 13:** Qualitative research results

Themes	Subthemes	Explanation	Quotes
Patient-Level Barriers to Self-Management	Weak self-management beliefs	Nearly half of the respondents expressed a lack of confidence in their ability to manage diabetes and prevent complications, particularly those already experiencing chronic complications (e.g., diabetic retinopathy, nephropathy) Contributing factors included poor self-control and reliance on family or community support In contrast, patients without complications and with satisfactory glycemic control reported stronger self-management beliefs	*Respondent 1: “I don’t feel confident about self-management or preventing complications. I already have macular edema and glaucoma*,* with partial visual loss.”* *Respondent 9: “I feel powerless to prevent complications. I can’t control myself and have to rely on doctors and nurses.”* *Respondent 3: “I believe I can control my blood sugar. My blood sugar is well managed so far*,* and I have no complications.”*
	Poor self-control and difficulty meeting self-management targets	Patients reported attempting lifestyle modifications (e.g., weight control, low-salt/low-fat diet, reduced sugar intake, glucose monitoring, regular medication, increased exercise) However, factors such as aging, memory decline, and polypharmacy impeded adherence While many patients reported post-meal walking to help control blood sugar, few met the recommended ≥ 150 min of moderate-intensity activity per week, and none engaged in resistance training	*Respondent 25: “I’m older now and sometimes forget to take my medicine. Missing a dose affects my condition.”* *Respondent 9: “It’s difficult to control my diet. I can’t always resist high-sugar or high-fat foods.”* *Respondent 23: “I usually walk 2*,*000–3*,*000 steps a day*,* but leg pain keeps me from walking further.”*
	Limited diabetes-related knowledge	Misconceptions about pharmacological treatment, fear of insulin, and concerns about side effects hindered optimal glycemic control	*Respondent 19: “My kidney function is declining. Some medications need dose adjustment. I’m afraid I’ll have to start insulin*,* and I’m worried it will harm my kidneys.”* *Respondent 16: “I don’t have accurate knowledge about medication management. I need doctors’ guidance.”*
Family-Level Barriers to Support	Lack of psychological support	Caregivers tended to focus on supervision of diet, medication, and exercise, but overlooked patients’ emotional needs for understanding and empathy	*Respondent 11: “I hope my family reminds me to take medicine*,* encourages me to exercise*,* and listens to me so I feel cared for.”* *Respondent 9: “Family members should communicate more*,* listen to my inner thoughts*,* give encouragement and comfort*,* and help me maintain a positive mindset.”* *Respondent 18: “I wish my family could understand the suffering of living with diabetes.”*
	Avoidance of family support	Some patients minimized the perceived risk of diabetes or avoided burdening their family, which reduced acceptance of support	*Respondent 20: “I can manage myself. My memory and vision are fine*,* so I don’t need help right now.”* *Respondent 18: “I can manage daily life on my own*,* and my pension is sufficient. For now*,* I don’t need additional help from my family*,* as everyone is quite busy.”* *Respondent 1: “My family members have already done a lot for me. I don’t want to trouble them more. I try to do things by myself.”*
	Predominantly supervisory support with limited participation	Caregivers often provided reminders or monitoring, but less active involvement in joint behaviors like exercise or meal planning	*Respondent 8: “My family urges me to take medicine*,* bought me a glucometer*,* and reminds me to exercise and improve my diet.”* *Respondent 22: “They remind me to take my medication regularly. It would be better if they exercised with me.”*
	Need for greater caregiver knowledge and emergency skills	Patients expected caregivers not only to share knowledge but also to recognize hypoglycemia and provide appropriate first aid	*Respondent 5: “I hope they understand diabetes better and know how to handle hypoglycemia emergencies.”*
Community-Level Barriers to Support	Increasing demand for individualized care	Although community health centers provided general education and lifestyle guidance, individualized interventions were lacking	*Respondent 22: “I hope the community can provide personalized dietary advice based on my condition.”*
	Need for long-term and accessible care	While periodic check-ups and screenings were available, continuity of care remained insufficient	*Respondent 5: “I expect regular follow-up from the community hospital*,* with timely blood glucose checks and communication.”*
	Inadequate medication supply	Limited availability or interruptions in essential diabetes medications affected disease control	*Respondent 18: “I hope the community pharmacy has a full supply and avoids stock-outs.”*
	Need for enhanced health education and training	Current educational activities (e.g., lectures) were not sufficient to meet patients’ learning needs	*Respondent 8: “Could the community organize more activities*,* lectures*,* or patient groups to learn about diabetes?”*

We employed Colaizzi’s seven-step method for qualitative data analysis. The specific procedures were as follows: (1) interview recordings were transcribed verbatim and repeatedly read to obtain an overall understanding; (2) significant statements related to the phenomenon under study were extracted; (3) the meanings of these statements were formulated and coded; (4) the formulated meanings were organized into clusters of themes; (5) an exhaustive description of the phenomenon was developed; (6) the fundamental structure of the phenomenon was further refined; (7) the results were returned to some participants for validation to ensure accuracy and trustworthiness.

To ensure the trustworthiness of the study, we adopted Lincoln and Guba’s evaluation criteria for qualitative research, including credibility, dependability, confirmability, and transferability. Specific strategies included repeated cross-checking of transcripts, peer debriefing and triangulation among researchers, member checking with participants, and maintaining detailed documentation of the research process and contextual information.

The quantitative phase provided a broad understanding of self-management behaviors and family support among patients with T2DM, while the qualitative phase explored the contextual and experiential dimensions underlying these patterns. Results from both components were independently analyzed and then compared through an integrative joint display, allowing cross-validation of findings and identification of convergent and complementary insights.

Quantitative data indicated deficiencies in diabetes knowledge and self-management behaviors, which were substantiated by qualitative themes highlighting weak self-management beliefs, poor self-control, and limited family and community support. Conversely, qualitative findings provided nuanced explanations for statistical trends, illustrating how psychological, familial, and community barriers jointly shaped patient behaviors. Discrepancies between quantitative and qualitative results were examined to refine interpretation and strengthen the theoretical depth of the study.

This triangulated approach ensured that conclusions were supported by multiple sources of evidence, thereby enhancing the robustness, credibility, and transferability of the results. Detailed findings are showed in Table 14.

**Table 14:** Integration of quantitative and qualitative findings (Methodological Triangulation)

Quantitative Findings	Qualitative Themes	Integrated Interpretation
Moderate overall KAP scores (77.56 ± 12.14) among T2DM patients; knowledge and practice were particularly weak.	Limited diabetes-related knowledge and misconceptions (*“I don’t have accurate knowledge about medication management.”*)	Consistent findings indicate insufficient understanding of disease management, emphasizing the need for targeted health education.
Patients showed low practice scores, especially in exercise (67.48 ± 29.85) and foot care (69.12 ± 21.33) dimensions.	Poor self-control and difficulty maintaining lifestyle changes (*“I can’t always resist high-sugar or high-fat foods.”*)	Both data sources reveal inadequate behavioral adherence; barriers relate to motivation, aging, and physical limitations.
Caregivers showed high attitude but moderate knowledge scores (83.25 ± 16.67).	Need for greater caregiver knowledge and emergency skills (*“I hope they understand diabetes better and know how to handle hypoglycemia.”*)	Quantitative and qualitative results converge, highlighting the need for caregiver training in knowledge and emergency management.
Patients and care givers both showed positive attitudes toward T2DM management (92.28 ± 13.36 and 95.28 ± 12.38) but low practice-performance gap.	Avoidance of family support (*“I can manage daily life on my own*,* and my pension is sufficient. For now*,* I don’t need additional help from my family*,* as everyone is quite busy.”*) Lack of psychological support (*“I wish my family could understand the suffering of living with diabetes.”*)	Quantitative–qualitative convergence shows that the positive attitude has not translated into effective behaviors, which may be attributed not only to a lack of basic knowledge and skills but also to intentional avoidance and insufficient psychological support.
-	Need for individualized, continuous, and accessible community care (*“I hope the community can provide personalized dietary advice.”*)	Qualitative data sets identify gaps in continuity and personalization of community services; interventions should enhance long-term, tailored support.

## Discussion

This study employed a mixed-methods design, combining a cross-sectional survey with qualitative interviews, to examine the self-management status of T2DM patients and their caregivers in Chaoyang District, Beijing, and to explore barriers at the patient, family, and community levels.

The findings showed that the surveyed population was mainly older adults with at least a high school education. However, elderly patients and those with lower educational levels had significantly lower KAP scores, consistent with previous studies [19]. This may be explained by decline in cognitive function among older T2DM individuals [20], low technology literacy, and limited ability to use self-management tools effectively [21]. For these groups, educational interventions should be simplified, use plain and accessible language, and incorporate visual materials and repeated reinforcement. Education should avoid heavy reliance on smartphones or other digital platforms that older patients may struggle to use.

Patients demonstrated relatively good knowledge of blood pressure and glucose control, suggesting some success of long-term education. Nonetheless, overall self-management remained inadequate, with notable deficits in basic knowledge, hypoglycemia self-rescue, exercise knowledge, and practice. Qualitative interviews reinforced these results, highlighting weak self-management beliefs, poor self-control, and limited understanding of treatment. Interventions aimed at improving health literacy have been shown to enhance glycemic control and self-management [22]. The study found that patients with T2DM had poor basic knowledge and several misconceptions, which is similar to findings reported in another developing country [23]. The combination of advanced age and a less connected information environment after retirement may result in fewer opportunities to access relevant knowledge. Low levels of diabetes-related knowledge are also an important risk factor affecting patients’ psychological well-being [24]. Notably, patients rarely integrated complication prevention into daily management, instead relying on periodic laboratory tests for follow-up. This suggests limited awareness of risk prevention and insufficient complication-related knowledge. Primary healthcare providers should therefore deliver targeted education on basic diabetes knowledge, exercise, hypoglycemia management, and complication screening, supported by lectures, peer discussion groups, and caregiver training. This may also be due to substantial gaps in knowledge and practice of Chinese primary care physicians [25]. Measures such as professional skills training, regular case discussions, technical supervision, and performance evaluations, for doctors and nurses in primary health care institutions, should be implemented to improve management standardization and participation [26].

Exercise therapy, as a cornerstone of non-pharmacological treatment, was poorly adhered to. In addition, limited knowledge of hypoglycemia and physical exercise constitutes another barrier to patients’ compliance with exercise recommendations [27]. Sarcopenia and T2DM have a bidirectional relationship [28]. Prior studies have shown that elderly T2DM patients have high rates of muscle mass loss and sarcopenia [29]. Meanwhile, studies have shown that patients with T2DM and sarcopenia have a higher mortality rate than those without sarcopenia [30]. Resistance exercise training is beneficial for enhancing glycemic control, lipid profiles, lean mass, and muscle strength in older adults with T2DM [31], yet most patients in this study lacked sufficient knowledge and did not exercise regularly. Tailored education on aerobic, resistance, hypoglycemia self-rescue and flexibility exercises is therefore urgently needed.

At the family level, caregivers scored higher than patients overall, but still demonstrated knowledge gaps, particularly in exercise, hypoglycemia management, and basic diabetes knowledge. Consequently, family support was often superficial, limited to reminders and supervision, without sufficient participation in shared management behaviors. In emergencies such as hypoglycemia, inadequate caregiver skills risked delays in care and destabilized glucose control. Active family involvement plays a positive role in patients’ disease management [10]. Targeted training for caregivers should therefore be incorporated into community health programs, teaching skills such as dietary recording, joint exercise planning, and hypoglycemia management [32]. Interviews also revealed limited caregiver psychological support. Depression is common in T2DM patients [33], and is linked to poor glycemic control [34]. Family function directly and indirectly influences loneliness through depression as a mediator [35]. However, some patients in this study reduced help-seeking due to concerns about burdening relatives, limiting family support and contributing to an “attitude–behavior gap.” Medical staff should therefore engage both patients and caregivers in emotional and psychological support training, encouraging empathy, open communication, and timely professional psychological care when needed.

At the community level, patients expressed a need for more individualized, long-term, and accessible management. Current programs provide general education and periodic screening but lack personalization and continuity. Community-based management can improve glycemic control and the achievement of treatment targets in patients with T2DM [36]. Telemedicine lifestyle support programs with personalized interventions have been shown to improve glycemic control [37]. Suggested strategies include establishing family health records, updating them regularly, and providing personalized educational materials and behavior guidance. Family doctors should adopt a bio-psycho-social model, tailoring intervention plans for diet, medication, and exercise. Implementation of family doctor contracts, with team-based continuous management, follow-up of irregular attendees via telephone or SMS, and mobile app–based remote monitoring may further optimize outcomes [38].

### Strengths and limitations

This study employed a mixed-methods design combining a cross-sectional survey and qualitative interviews to comprehensively examine the self-management status of patients with T2DM in Chaoyang District, Beijing, as well as the challenges related to family and community support. The integration of quantitative and qualitative findings through methodological triangulation enhanced the credibility and interpretive depth of the results. The study’s strengths include diverse sampling sources, reliable measurement tools, and rigorous qualitative analysis using Colaizzi’s method, with results integrated via a joint display to demonstrate convergence and complementarity between data types. Nonetheless, several limitations should be acknowledged. The study sample was confined to Chaoyang District, which may limit generalizability; the cross-sectional design precludes causal inference; self-reported data may be subject to recall and social desirability bias; and the qualitative sample was relatively small and drawn from a single site. Future multicenter and longitudinal mixed-methods studies are warranted to validate and extend these findings.

## Conclusion

This study identified significant gaps in knowledge and practice among T2DM patients in Chaoyang District, with particularly low adherence to key self-management behaviors. Caregivers performed better overall but also demonstrated areas needing improvement. Barriers at multiple levels may compromise glycemic control and hinder achievement of management goals.

To enhance diabetes management in community settings, efforts should focus on patient education, family involvement, and community capacity building. Patient education should aim at improving disease awareness and self-management skills, covering key topics such as complications, exercise and foot care. Diverse formats—including lectures, group discussions, and online learning—can increase engagement and accessibility. Family education programs are also essential to improve family members’ understanding of diabetes and their ability to provide effective support. Training should address basic knowledge, complication prevention, and psychological support through lectures, case studies, and role-playing. At the community level, greater investment is needed to strengthen health service centers by expanding medical equipment, drug supply, and staffing. Continuous training for community physicians and the development of an integrated diabetes management information system can facilitate information sharing and personalized, long-term care.

## Data Availability

Data can be obtained from the corresponding author upon reasonable request.

## Abbreviations

T2DM: Type 2 Diabetes Mellitus
KAP scale: Knowledge, Attitude, and Practice scale
